# Spinal cord vascular degeneration impairs duloxetine penetration

**DOI:** 10.3389/fpain.2023.1190440

**Published:** 2023-05-26

**Authors:** M. E Da Vitoria Lobo, R Madden, S Liddell, M Hirashima, R. P Hulse

**Affiliations:** ^1^ Cancer Biology, Division of Cancer and Stem Cells, School of Medicine University of Nottingham, Nottingham, United Kingdom; ^2^ Exonate Ltd., Nottingham, United Kingdom; ^3^ Division of Pharmacology, Niigata University Graduate School of Medical and Dental Sciences, Niigata, Japan; ^4^ School of Science and Technology, Nottingham Trent University, Nottingham, United Kingdom

**Keywords:** pain, analgesia, duloxetine (DLX), endothelial, spinal cord, VEGF—vascular endothelial growth factor, permeability

## Abstract

**Introduction:**

Chronic pain is a prevalent physically debilitating health-related morbidity. Frontline analgesics are inadequate, providing only partial pain relief in only a proportion of the patient cohort. Here, we explore whether alterations in spinal cord vascular perfusion are a factor in reducing the analgesic capability of the noradrenaline reuptake inhibitor, duloxetine.

**Method:**

An established rodent model of spinal cord vascular degeneration was used. Endothelial-specific vascular endothelial growth factor receptor 2 knockout mouse was induced via hydroxytamoxifen administered via intrathecal injection. Duloxetine was administered via intraperitoneal injection, and nociceptive behavioural testing was performed in both WT and VEGFR2KO mice. LC-MS/MS was performed to explore the accumulation of duloxetine in the spinal cord in WT and VEGFR2KO mice.

**Results:**

Spinal cord vascular degeneration leads to heat hypersensitivity and a decline in capillary perfusion. The integrity of noradrenergic projections (dopa - hydroxylase labelled) in the dorsal horn remained unaltered in WT and VEGFR2KO mice. There was an association between dorsal horn blood flow with the abundance of accumulated duloxetine in the spinal cord and analgesic capacity. In VEGFR2KO mice, the abundance of duloxetine in the lumbar spinal cord was reduced and was correlated with reduced anti-nociceptive capability of duloxetine.

**Discussion:**

Here, we show that an impaired vascular network in the spinal cord impairs the anti-nociceptive action of duloxetine. This highlights that the spinal cord vascular network is crucial to maintaining the efficacy of analgesics to provide pain relief.

## Introduction

Pain is inherently associated by all organisms as an uncomfortable but fundamentally integral physiological mechanism that protects tissues from damage or lasting harm. However, chronic pain is one of the leading causes of morbidity globally, represented by 26 million people in the United Kingdom (1) and 1.9 billion people worldwide (2) who are affected by this condition. Furthermore, a number of distinctly diverse causative factors [such as age (3), familial/genetic (4), disease (5), or treatment (6)] cause this physical affliction. Chronic pain is depicted as long-lasting inescapable pain that can be presented in a number of physical forms with exacerbated sensory perception highlighted by heightened sensations to evoked stimuli as well as ongoing pain. Furthermore, chronic pain is a significant psychophysical burden, which greatly impacts upon an individual’s quality of life (7). Current pain management regimens used clinically encompass the utilisation of wide-ranging groupings of differing pharmacological agents. Primary analgesics typically prescribed to diabetic neuropathic pain patients are non-steroidal anti-inflammatory drugs (8) and serotonin (5HT)/noradrenaline (NA) reuptake inhibitors (SNRIs) (9). Pregabalin and duloxetine (9) are widely recognised SNRIs and used in the treatment of diabetic neuropathic pain (NICE approved primary analgesics) (10). These act by dampening down the aspects of the central nervous system (CNS) deemed to be hyperexcitable during pathological pain states (11). As an SNRI, duloxetine enhances endogenous pain control through increasing 5HT and/or NA availability in the spinal cord to drive endogenous analgesic actions (12, 13). Despite this recommendation, current use of these analgesics only provides partial relief from symptoms in the majority of cases, e.g., diabetic neuropathy patients get little to no pain relief (up to ∼61% of patients) (9, 14–16). Furthermore, increasing proportions of diabetic neuropathic pain patients discontinue the use of the prescribed analgesic (duloxetine or pregabalin) due to poor treatment efficacy or adverse health-related side effects (15, 17). This is increasingly apparent for duloxetine use (17), with 86% of patients discontinuing treatment use 4 weeks following initial use (9, 15).

Despite extensive investigations into the underlying cellular and molecular mechanisms of diabetic neuropathic pain, availability of disease-tailored analgesics, in particular, for diabetic sensory neuropathy remains elusive (5). Recent evidence has implicated the blood–spinal cord barrier (BSCB) as a fundamental mediator of chronic pain with enhanced vascular permeability underlying chronic inflammatory pain (18–20). This is depicted by the infiltration of inflammatory cell types and consequent upregulation of inflammatory mediators locally in the somatosensory nervous system (21). However, further evidence has highlighted opposing actions in relation to the blood–spinal cord barrier, with curtailed blood flow and degeneration of the spinal cord microvasculature initiating neuropathic pain states (19, 22). This study aims to elucidate whether a reduction in the spinal cord microvasculature will lead to reduced accumulation of duloxetine in the spinal cord. We demonstrate that in a previous described rodent model of vascular degeneration (19, 22), there are decreased levels of duloxetine penetrating the spinal cord and consequently a reduction in duloxetine induced alleviation of pain hypersensitivity.

### Ethical approval and animals used

**Figure 1:**
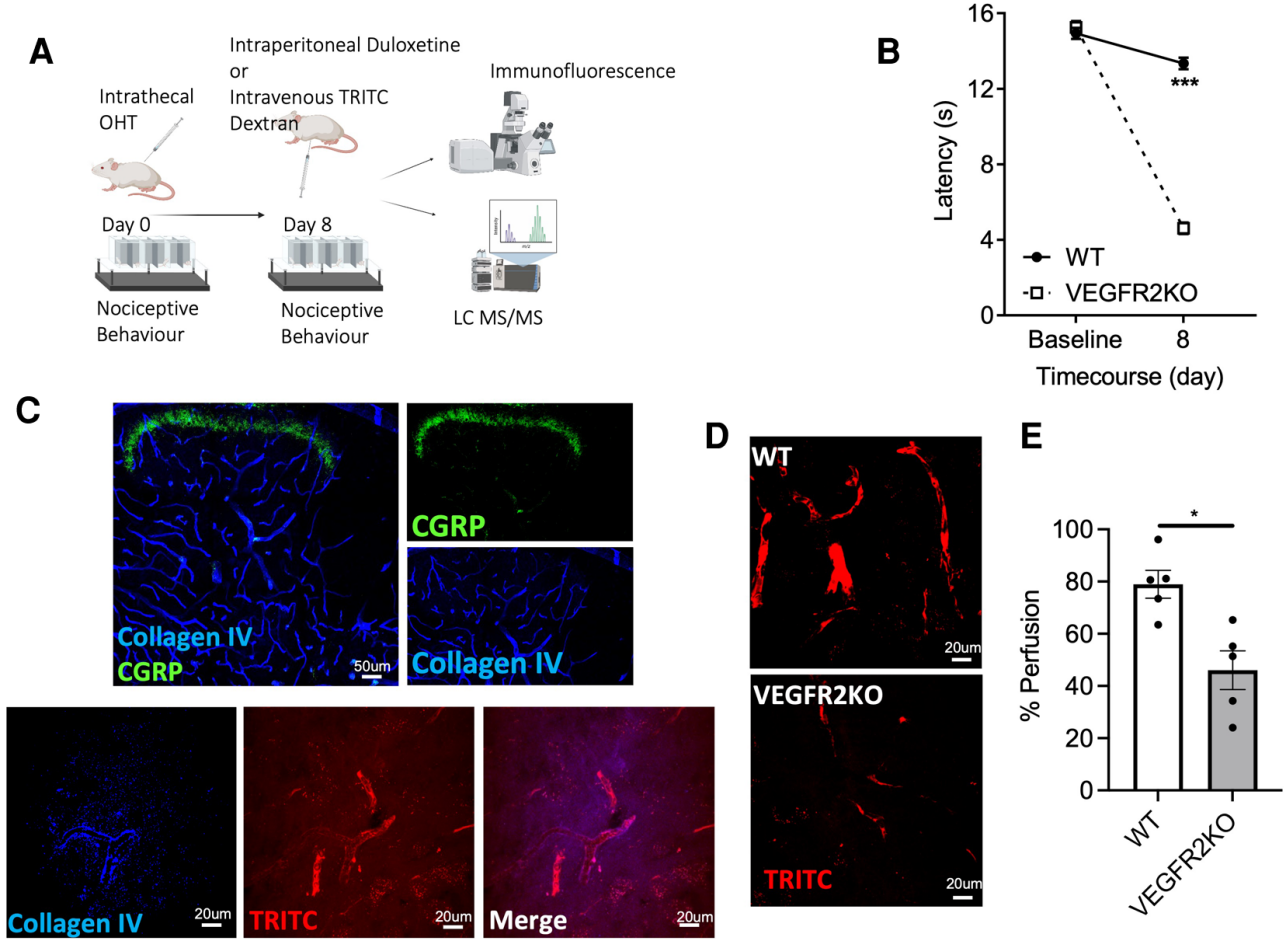
A transgenic rodent model of vasculopathy. (**A**) Experimental timeline is outlined in the diagrammatic figure. Both VEGFR2KO (Tie2CreER^T2^ *positive* x vegfr2^fl/fl^) mice and WT (Tie2CreER^T2^ *negative* x vegfr2^fl/fl^) mice were administered with hydroxytamoxifen (1 µM) via intrathecal injection. (**B**) 8 days post hydroxytamoxifen injection, a pronounced heat hypersensitivity in VEGFR2KO mice developed. This was demonstrated through a decrease in withdrawal latency when compared to WT mice (***P < 0.001, two-way ANOVA with post-Sidak multiple comparison test, *N* = 8 WT, *N* = 10 VEGFR2KO; WT mean ± SEM: 13.14 ± 0.37 s vs. VEGFR2KO mean ± SEM: 4.61 ± 0.30 s). (**C**) Representative images of capillary network in the mouse lumbar dorsal horn. (**C**, upper panel) Collagen IV (basement membrane marker) and superficial dorsal horn marker (CGRP) demonstrates the extensive vascular network throughout the dorsal horn. (**C**, Lower Panel) TRITC (Red) perfused lumbar spinal cord cryosections stained with basement membrane marker (collagen IV; Blue). (**D**) There is diminished capillary perfusion in VEGFR2KO mice vs. controls, presented as a reduction in the percentage of (**E**) TRITC dextran perfused vessels in VEGFR2KO mice vs. controls (*P < 0.05, Paired *T* test, *N* = 5 WT, *N* = 5 VEGFR2KO).

Animal studies were performed in accordance with the ARRIVE guidelines, with experimental protocols reviewed by the local Animal Welfare and Ethics Review Board (University of Nottingham). All studies were performed under UK Home Office animals (Scientific procedures) Act 1986 and EU Directive 2010/63/EU. Animals had *ad libitum* access to standard chow and were housed in groups under 12:12 h light:dark conditions. Experimental timeline are outlined in Figure 1A.

### Transgenic mouse model

An endothelial-specific promoter-driven Cre recombinase mouse line was used [Tie2CreER^T2^ mice; European Mutant Mouse Archive Tg(Tek-cre/ER^T2^)1Arnd] and crossed with homozygous *vegfr2*^fl/fl^ mice (19, 22). The induction of the transgenic model is previously described and does not impact upon the surrounding tissues, i.e., dorsal root ganglia (19, 22). Mice were 8 weeks old and of both genders. All the mice (total 39) were vegfr2^fl/fl^ positive and either Tie2CreER^T2^ negative (*n* = 24, termed WT) or positive (*n* = 25, termed VEGFR2KO). All mice were briefly anaesthetised using isoflurane (∼2% O_2_) to allow a single 10 μl intrathecal injection of 1 µM 4-hydroxytamoxifen (OHT; 10% ethanol in sunflower oil) between lumbar vertebra 5 and 6 (19). Studies were performed 8 days post OHT injection, a time point in which overt pain behaviours were presented.

### Nociceptive behaviour assays

All animals were habituated to the experimental environment and handling. Nociceptive behavioural assays were performed pre and post OHT administration, followed by before and after duloxetine injection. Thermal hyperalgesia is measured using a radiant heat source directed against the plantar surface of the hind paws through a Perspex floor (23), and the latency to withdrawal is measured. The stimulus intensity is determined at the beginning of each experimental series to give a control withdrawal latency of ∼10 s. This intensity is subsequently used for the rest of the nociceptive behavioural study. A maximum latency duration of 20 s is used to prevent tissue damage/sensitization due prolonged exposure to intense sustained stimulation. The mean withdrawal latency is determined from three repeated stimulations at an interstimulus interval of at least 3 min.

### Immunohistochemistry

Rodents were terminally anaesthetised (i.p., 60 mg/kg sodium pentobarbital) and tetramethylrhodamine isothiocyanate (TRITC; 1 mg/20 g rodent) dextran (76 Mw; Sigma-Aldrich) was intravenously administered. Mice were subsequently transcardially perfused with phosphate buffered saline (PBS) followed by 4% paraformaldehyde (PFA; in PBS pH7.4). The vertebral column was excised and the lumbar region of the spinal cord was extracted. The lumbar spinal cord was submerged in 4% PFA overnight (4°C). Samples were subsequently submerged in a 30% sucrose solution overnight (4°C). Prepared spinal cords were embedded and frozen in OCT. These samples were stored at −80°C until required for cryosection processing. Spinal cords were cryosectioned (40 µm thickness) and mounted on Superfrost plus slides (VWR). Slides were washed with PBS and incubated in blocking solution (5% bovine serum albumin and 10% fetal bovine serum in PBS 0.2% Triton X-100) for 1 h at room temperature. Slides were further incubated in a primary antibody [rabbit anti-collagen IV, 1 in 200 Abcam; goat anti-CGRP, 1 in 500 Abcam; or rabbit anti-DβH (dopamine β-hydroxylase), 1 in 500; Millipore, AB1538] in a blocking solution (overnight 4°C). Slides were washed with PBS and secondary antibodies were applied (PBS, 0.2% Triton X-100; 1 in 500 Alexa Fluor 488-conjugated donkey anti-mouse or Alexa Fluor 405-conjugated donkey anti-rabbit or goat, Thermofisher, United Kingdom). Slides were incubated in secondary antibody solutions at room temperature for 2 h. Vectashield mounting media (Vectorlabs) were used to mount the coverslips. Spinal cord sections were imaged using a confocal microscope (Leica, SP5). Immunohistochemistry of dorsal horn (DH) neuronal projections in the lumbar spinal cord were quantified from random nonsequential sections, with integrated density (i.e., the fluorescence intensity of DH immunoreactivity) and % area of DβH fluorescent staining across the dorsal horn determined. Blood vessel perfusion was determined as the ratio between collagen IV and TRITC dextran.

### LC-MS/MS sample preparation

Eight days post OHT administration, a time point in which pain and vascular degeneration manifest, duloxetine (DLX; 20 mg/kg) was administered via a single intraperitoneal injection. Nociceptive behavioural testing was performed 30 min after administration of duloxetine, and consequent to this, the lumbar spinal cord was extracted, frozen, and stored at −80°C until tissue processing. Lumbar spinal cord tissues were weighed prior to processing and homogenised in ultrapure water using a microtube homogeniser (Bead Bug; Benchmark Scientific); methanol was added to the homogenate and spiked with the internal standard to achieve a final concentration of 3 pg/µl. The precipitated solution was stored at −20°C before LC-MS/MS.

### LC-MS/MS

The quantity of duloxetine in the spinal cord samples was determined using liquid chromatography-mass spectrometry (24). Duloxetine (100 pg/µl) and the internal standard (30 pg/µl) were prepared in methanol and a calibration curve for duloxetine was generated to determine a signal intensity response ratio between duloxetine and internal standard (IS). Duloxetine calibration curve was prepared in 150 µl of methanol, with 5 µl of 30 pg/µl IS, and 145 µl methanol added. These points were then added to 450 µl of methanol within a precipitation filter plate. Quality control (QC) samples were prepared at concentrations of 6.25 pg/μl (High), 0.390625 pg/μl (middle), and 0.02441406 pg/μl (lower limit of quantitation). The calibration and QC samples were stored at −20°C until analysis and performed in duplicate. A Nexera X2 HPLC system (Shimadzu) fitted with a Kinetex EVO C18 100 Å LC column (50 mm × 2.1 mm, 5 µm; Phenomenex), a binary LC-30AD prominence pump, an autosampler (SIL-30AC), and a solvent degasser (DGU-20A_3R/5R_) were used for this study. An LCMS-8050 liquid chromatography-mass spectrometer (Shimadzu) equipped with electrospray ionisation (ESI) interface at 300°C was employed for the analyte and IS detection. MRM mode was employed for ion detection through monitoring the transitions of duloxetine and the IS. (S)-Duloxetine hydrochloride (Sigma Aldrich) was utilised, with Duloxetine D3-HCl (Sigma Aldrich) as an internal standard. Five-microliter aliquots of samples were injected into the column and maintained at 40°C. Two isocratic mobile phases consisting of either 30% (Mobile phase A) or 90% (Mobile phase B) methanol (Fisher Scientific) and 20 mM ammonium formate (pH 8.5; Honeywell Research Chemicals) were employed to separate the analyte from the endogenous components through a flow rate of 0.6 ml/min and a retention time of 0.79 min into the electrospray ionisation chamber of the mass spectrometer. The precursor ion for duloxetine had an *m*/*z* of 298.10 with transition ions (products/fragments) of *m*/*z* 44.20 and 154.25. The precursor ion for the IS had an *m*/*z* of 301.10 with transition ions of *m*/*z* 47.25 and 157.15. Quadrupoles Q1 and Q3 were set to unit resolution. The collected analysis data were processed using Lab Solutions (Shimadzu).

### Statistical analysis

All data are represented as mean ± SEM unless stated, with WT control animals compared to VEGFR2KO animals. Samples sizes were performed using G power using historical nociceptive behavioural datasets utilised (18, 19, 22). Data were analysed using Microsoft Excel and Graphpad Prism 8. A Two-way ANOVA with post-Sidak test was used for statistical analysis of nociceptive behavioural data. Statistical analysis was performed using a Mann–Whitney test. Immunohistological analysis was performed on nonsequential sections of the lumbar dorsal horn with a minimum of five sections per animal analysed and an average acquired per animal. The ratio of signal response for duloxetine against IS was compared against the calibration curve to determine the unknown concentration of duloxetine within the tissue. These values from each experimental group were compared using a Mann–Whitney test, with comparison to nociceptive withdrawal latency determined using a linear regression analysis against WT and VEGFR2KO values. No animals were withdrawn or data points were removed from this study. Diagrammatic figure was created in Biorender.com.

## Results

### Vascular degeneration in the spinal cord causes diminished duloxetine accumulation

An established spatiotemporal model of vascular degeneration that leads to a pronounced heat hyperalgesia was used in this study. The model was induced via an intrathecal delivery of OHT to induce an endothelial-specific vascular endothelial growth factor receptor 2 (VEGFR2) knockout mouse model (19, 22). Eight days post-intrathecal administration of hydroxytamoxifen in Tie2CreER^T2^-positive-*vegfr2*^fl/fl^ mice (VEGFR2KO) results in heat hypersensitivity (Figure 1B, *P* < 0.001), when compared to Tie2CreER^T2^-negative −*vegfr2*^fl/fl^ mice (WT). TRITC dextran is a fluorescent tracer and when delivered intravenously enables the identification of a perfused capillary. Dorsal horn (Figure 1C representative image of lumbar dorsal horn–capillary network and nociceptive inputs) vessels labelled with collagen IV and positive for tritc dextran were deemed to be a perfused spinal cord capillary (representative images in Figures 1C,D), with vessels presenting less TRITC dextran are non-perfused. Eight days post OHT intrathecal administration, there was a reduction in lumbar spinal cord capillary perfusion in the lumbar region of the spinal cord in VEGFR2KO vs. control rodents (Figure 1E, *P* < 0.05).

**Figure 2:**
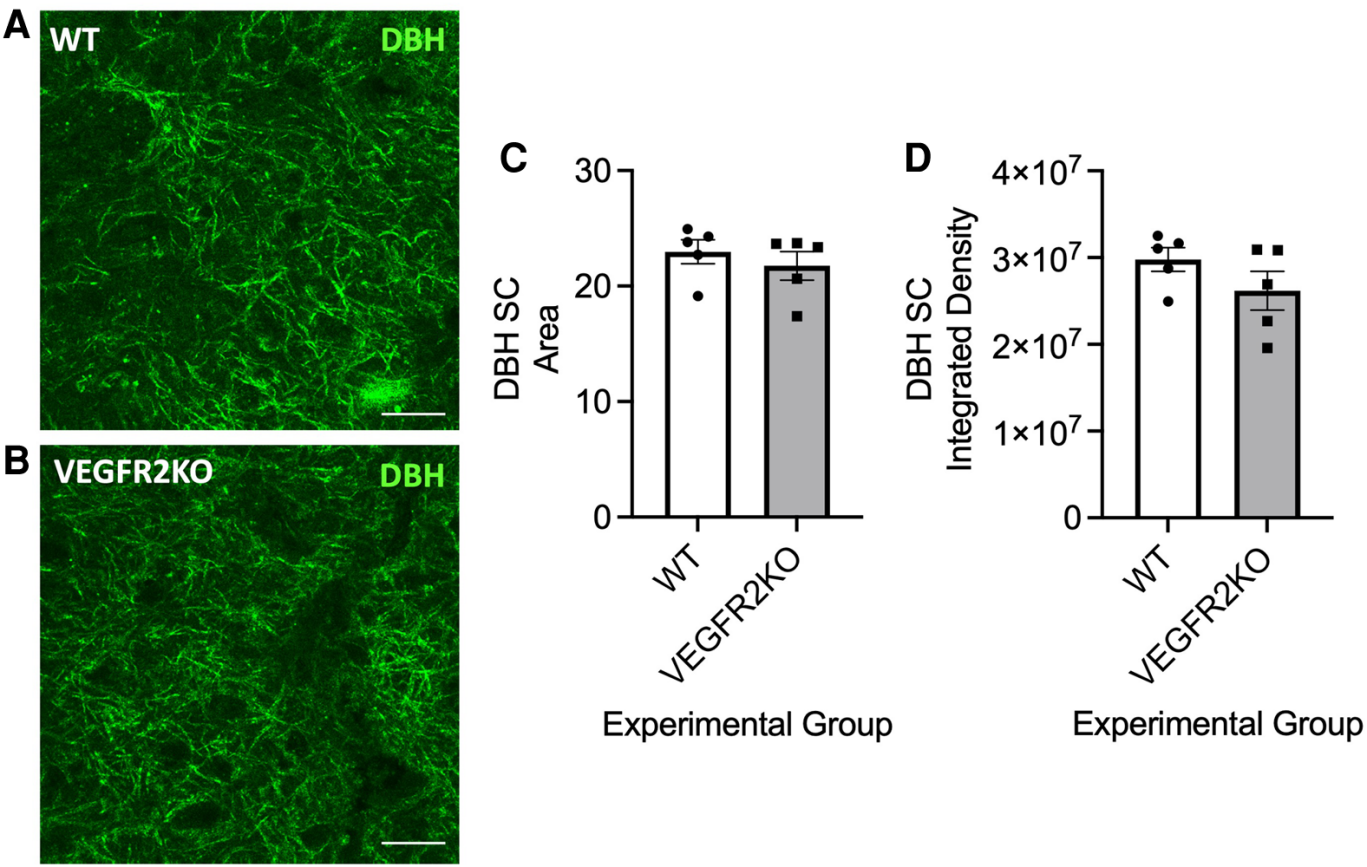
In a transgenic rodent model of vasculopathy-induced pain, noradrenergic fibre innervation into the dorsal is unchanged. Representative image examples of noradrenergic DβH-positive processes in the lumbar dorsal horn of (**A**) WT and (**B**) VEGFR2KO mice (scale bar = 10 µm). There was no change in DβH immunoreactivity within the dorsal horn [(**C**) area, WT mean ± SEM: 22.74 ± 0.99 A.U. vs VEGFR2KO mean ± SEM: 21.26 ± 1.410 A.U.; (**D**) integrated density, *N* = 5 per group].

Duloxetine is used in frontline treatment for neuropathic pain, in particular diabetic neuropathic pain. However, the efficacy is poor with few patients having benefit. Due to the cessated perfusion of the spinal cord vasculature in this model of vascular degeneration, we explored whether an impaired spinal cord capillary network impacts upon duloxetine penetration and accumulation in the CNS, in particular, the spinal cord. Duloxetine is a serotonin noradrenaline reuptake inhibitor, and previously it has been documented that noradrenergic (DβH-positive) projections in the spinal cord and noradrenergic dependent anti-nociceptive endogenous tone can be disturbed in models of chronic pain (13). Therefore, it was determined whether spinal cord DβH immunoreactivity was compromised in this transgenic mouse model. DβH immunoreactivity in the lumbar dorsal horn of VEGFR2KO mice was unchanged compared to WT littermates [high power representative dorsal horn images from WT (Figure 2A) and VEGFR2KO (Figure 2B) mice] as depicted by no change in DβH immunoreactivity in the dorsal horn (Figure 2C—DβH area; Figure 2D—DβH integrated density) of either WT or VEGFR2KO mice.

**Figure 3:**
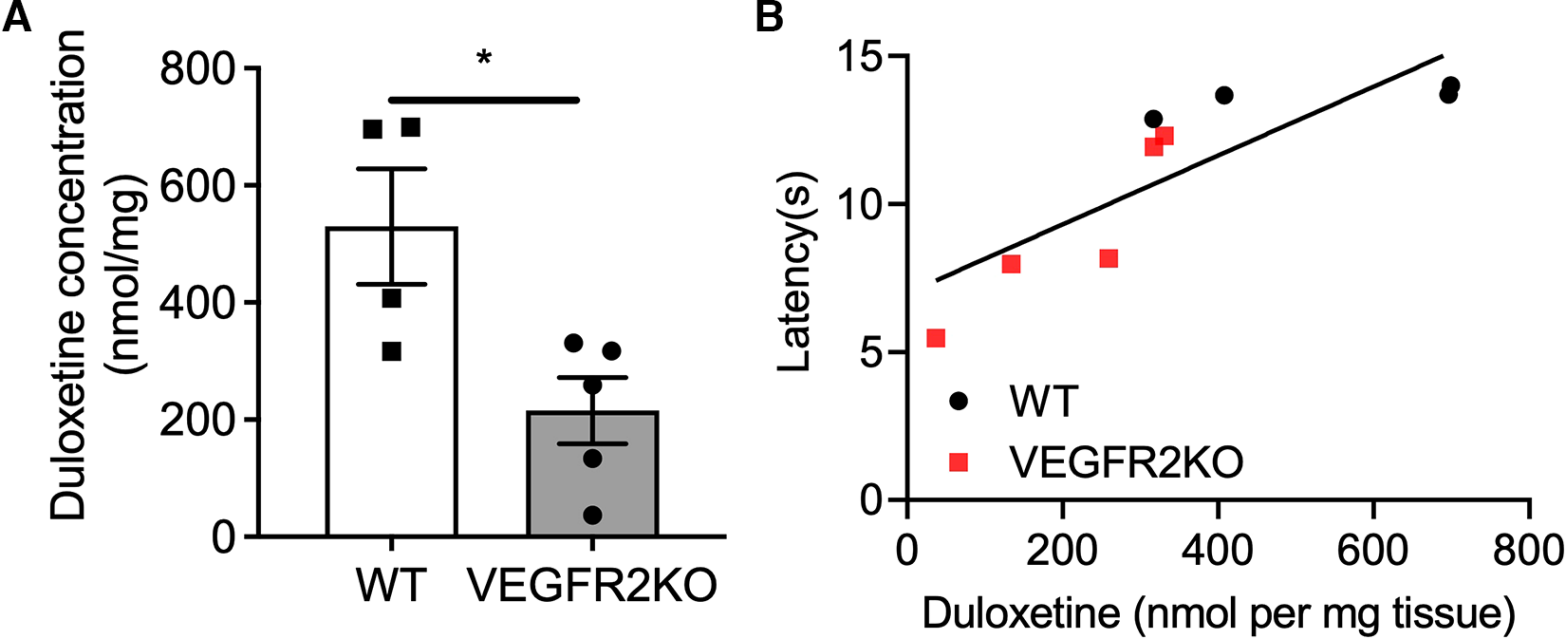
Duloxetine accumulation in the spinal cord is diminished in VEGFR2KO mice and impairs analgesia. 8 days following hydroxytamoxifen treatment in WT and VEGFR2KO mice, all mice received a single intraperitoneal injection of duloxetine (20 mg/kg). (**A**) The lumbar region of the spinal cord was excised and abundance of duloxetine was determined using LC-MS/MS. There was a decrease in duloxetine concentration in the spinal cord of VEGFR2KO mice when compared to WT mice [*P < 0.05, Mann–Whitney Test, *N* = 4 WT (mean ± SEM: 519.7 ± 98.63), *N* = 5 VEGFR2KO (mean ± SEM: 215.7 ± 56.64)]. (**B**) Correlation analysis of duloxetine abundance vs. heat withdrawal latency demonstrates a strong correlation (*R*^2 ^= 0.702, *P* < 0.01, single linear regression analysis) between duloxetine abundance and heat withdrawal latency. This demonstrates that those rodents with lowest abundance of duloxetine in the lumbar region of the spinal cord presented a lower withdrawal latency following administration (*P < 0.05, Paired *T* test, *N* = 4 WT, *N* = 5 VEGFR2KO).

To determine the impact of spinal cord vascular degeneration upon duloxetine accumulation in the spinal cord, quantitative analysis of duloxetine penetration and accumulation into the lumbar spinal cord was determined in WT and VEGFR2KO mice. The abundance of duloxetine that accumulated in the spinal cord of VEGFR2KO mice was significantly reduced compared to WT mice (Figure 3A, *P* < 0.05). In addition, there was a significant correlation between the concentration of duloxetine in the spinal cord vs. the heat withdrawal latency of the rodent (Figure 3B, *R* = 0.7). These data depict that those rodents with lower heat withdrawal latencies following DLX administration also were found to have lower accumulated duloxetine in the spinal cord depicting reduced DLX analgesia. These were found to be VEGFR2KO mice. However, conversely those WT rodents with higher withdrawal latencies post DLX treatment were found to have the highest accumulate duloxetine concentration.

## Discussion

Current technological advances and drug discovery programs are identifying novel analgesics that could curtail neuropathic pain (25), in a disease-specific manner (10). However, to date, there are few effective analgesics currently available due to limited measures of efficacy and tolerability in patients. This includes the frontline analgesic duloxetine that only benefits a proportion of neuropathic pain patients, in particular, diabetic neuropathic pain patients. This study presents an underlying pathophysiological process that contributes to the minimal impact that duloxetine has on a cohort of diabetic neuropathic pain patients. Here, we utilise a previously established model of vascular degeneration-induced heat hypersensitivity. Our previous work has demonstrated in a rodent model of chronic pain that nociceptive behavioural hypersensitivity is accompanied by a reduced structural and functional presence in the dorsal horn microvessel network. The data presented here highlight that this diminished microvascular network in the spinal cord impedes analgesic efficacy through reduced penetration of duloxetine into the spinal cord.

### Role of noradrenergic descending control of neuropathic pain

Monoamine reuptake inhibitors are primary frontline analgesics, as this classification of drugs has been proven to be effective in treating neuropathic pain (10). In particular, duloxetine along with pregabalin are recommended for the treatment of diabetic neuropathic pain (26, 27). The premise for the efficacy of monoamine reuptake inhibitors develops from the understanding that serotoninergic and noradrenergic descending processes mediate inhibitory modulation upon spinal cord neuronal activity (28). Pharmacological intervention through noradrenergic targeted reuptake inhibition results in the potentiation of these predominating inhibitory descending control actions derived from noradrenaline enriched neurons in the ponto-spinal circuitry (e.g., locus coereuleus) (29, 30). The utilisation of reuptake inhibitors (such as duloxetine) in naive situations demonstrates minimal effectiveness with no change typically presented in nociceptive behavioural paradigms (13, 31, 32). A lack of duloxetine activity is presented here in a vasculopathy-induced chronic pain model that induces only heat hyperalgesia (19, 22), where duloxetine does not alter nociceptive withdrawal thresholds in the control rodents. To note, it has previously been explored that naive transgenic VEGFR2KO mice do not present mechanical hypersensitivity (18). Vascular endothelial growth factor A (VEGF) signalling is pro-angiogenic, driving blood vessel maintenance, growth, and permeability. These actions are dependent upon VEGFR2 signalling and as previously presented, the loss of VEGFR2 signalling results in a decline in endothelial health and diminished capillary integrity. Previously, systemic and intrathecal induction of an endothelial-specific VEGFR2KO has only presented heat hyperalgesia (19, 22). However, in chronic pain rodent models, mechanical hypersensitivity was influenced in VEGFR2KO mice (18). Therefore, in states of chronic pain, duloxetine may present alternative nociceptive behavioural outcomes due to the perceived involvement of VEGFR2 in chronic pain manifestation.

Importantly, in neuropathic pain cohorts, an increase in noradrenergic inhibitory tone through the utilisation of reuptake inhibitors alleviates neuropathic pain phenotypes (13, 32). However, there is conjecture around the role and effectiveness of noradrenergic reuptake as a possible target for analgesia development, as only one patient in five gains therapeutic benefit (26). In contrast to the perceived dogma of the monoamine hypothesis, in some studies, there is an increase in the prevalence of dorsal horn DβH-positive fibres, which is accompanied by an equivalent increase in noradrenaline in the spinal cord in neuropathic pain states (13). Furthermore, in some investigations, there is no change in expression profiles of noradrenergic processes (33), similarly as shown in this study. Here, we present a lack of duloxetine activity due to reduced penetrance into the spinal cord and not due to a decline of noradrenergic processes. However, it is worth considering that other factors may be in play. A decline in the dorsal horn vascular network and subsequent reduction in oxygenation of the tissue, as a consequence, could influence the activity of the noradrenergic system. Considering that alternative rodent models of chronic pain demonstrate alterations in the actions of noradrenaline, it could be that a hypoxic environment could promote alterations in the synaptic handling of noradrenaline rather than the structural aspects of the noradrenergic projections. However, the overriding consensus is that studies exploring reuptake inhibitor treatment demonstrate pronounced analgesia following treatment, and accompanying increase in the expression of the noradrenergic signalling cascades (33, 34).

### Vasculopathy impairs analgesic delivery

In relation to the utilisation of reuptake inhibitors in the clinic, there is an inherent reliance upon these agents due to the partial alleviation of neuropathic pain, in particular, diabetic neuropathic pain. However, wider use and long-term utilisation are limited due to the inconsistent nature of effectiveness in pain relief. Determination of the processes that underlie this clinical problem is crucial to enable effective treatment to provide pain relief. One aspect of effective drug delivery is appreciation of the distribution system widely utilised in the body, the capillary network. The microvascular network provides an enormous delivery network through the expansive luminal surface area to supply tissues throughout the body with essential nutrients, to support efficient and integral physiological performance. However, drug distribution is also a key consideration. The blood–brain barrier (BBB) or, in this instance, the BSCB is a highly fundamental system that protects our bodies and, in particular, the CNS. It is widely appreciated the BBB or BSCB is highly impenetrable due to the reduced interjunctional proteins and fenestrations (35). This is accompanied by an extensive array of mural cells that surround the endothelial lumen to further modulate the integrity of the endothelium, while also contributing to the functional role (36). The BSCB also expresses channels and carrier proteins that control key processes that control the movement of specific agents and nutrients to pass across this barrier (37, 38). As a consequence, only a small proportion of agents are able to pass across this membrane into the neural tissues (39) and prevent foreign bodies such as pathogens from entering. This highly controlled accessibility also impacts upon pharmacological agents. This is a significant obstacle when developing novel treatments that target CNS-based diseases (40). However, during ageing and disease, these microvessel structures are damaged, such as in multiple sclerosis (36, 41, 42). These vessels typically develop increased permeability, a process that enables foreign entities to access the central nervous system (43). This leads to the initiation of inflammatory processes in the CNS due to the penetration of inflammatory cell types that cause harm to these previously protected neural structures (20, 21, 39). Our previous work has identified an opposing neuropathology that the microvessel network within the spinal cord can be damaged, in particular, with relation to diabetic neuropathic pain. A reduction in the dorsal horn endothelium is a factor in the onset of diabetic neuropathic pain in rodent models (19, 22). This deterioration of the blood vessel lumen leads to a reduction in spinal cord blood perfusion, demonstrated by decreased delivery of varying experimental solutes (19, 22). In this study, duloxetine alleviates heat hypersensitivity solely in the VEGFR2KO rodents. However, as stated in this model, there is a lack of vascular perfusion of the spinal cord, and also as stated here, in neuropathic pain patients there is a prominent lack of analgesic efficacy. We have explored the association of these factors here, with the total quantity of duloxetine reaching the spinal cord reduced in the VEGFR2KO rodents. Furthermore, a lower quantity of duloxetine present in the spinal cord is accompanied by a highly prevalent and persistent pain phenotype. This is of significant importance. These data demonstrate that a damaged BSCB endothelium leads to a reduced delivery of a pharmacological agent. Fundamentally, this impaired microvascular delivery system prevents analgesics reaching the required site of action, the central nervous system, and here the spinal cord, therefore negating the effect of providing analgesics to treat pain. However, it must be noted that duloxetine acts as a molecular tracer to allow the measurement of tissue perfusion in these studies. As a consequence in the unaffected, duloxetine responding animals, the spinal cord capillary network is expected to be maintained as duloxetine abundance is at higher levels and provides an analgesic action.

Here, we provide an understanding of why pain relief for neuropathic pain patients is in part ineffective in a proportion of pain patients. However, to note, analgesics would still be expected to engage with the peripheral sensory nervous system as well as supraspinal regions. The delivery of a gold standard analgesic, duloxetine, is impaired in a rodent with chronic pain that develops due to vasculopathy of the spinal cord. As a result, drug discovery programmes should consider disease-tailored factors when designing analgesia. Additionally, the analgesia delivery regimen needs to be considered when treating pain patients to allow these agents to be delivered effectively to the required site of action required.

## Data Availability

The raw data supporting the conclusions of this article will be made available by the authors, without undue reservation.
